# Complex Venous Thromboembolism in a Young Woman With Multisystem Manifestations: A Case Report

**DOI:** 10.7759/cureus.92328

**Published:** 2025-09-14

**Authors:** Mahmoud A Alswij, Sliman Marina, Mais Musleh, Dani Alokla, Adnan Alhaj Hasan

**Affiliations:** 1 Hematology, Al Mouwassat University Hospital, Damascus, SYR; 2 Gynecology, Damascus University, Damascus, SYR; 3 Emergency, Damascus Hospital, Damascus, SYR; 4 Internal Medicine, Al Mouwassat University Hospital, Damascus, SYR

**Keywords:** anticoagulation, cerebral venous sinus thrombosis, mesenteric venous thrombosis, sickle cell trait, thrombophilia, venous thromboembolism

## Abstract

Venous thromboembolism (VTE) in adolescents is rare and often necessitates a comprehensive evaluation for both inherited and acquired prothrombotic conditions. We report the case of an 18-year-old female who initially presented with cerebral venous sinus thrombosis (CVST). Anticoagulation with warfarin was complicated by massive gastrointestinal bleeding, followed by deep vein thrombosis (DVT) and recurrent thrombotic events, including superior mesenteric venous thrombosis with bowel infarction. Laboratory investigations revealed sickle cell trait (HbS 38.3%), heterozygous mutations in lymphotoxin alpha (LTA C804A), Factor V R2 (H1299R), and methylenetetrahydrofolate reductase (MTHFR C677T), with normal homocysteine levels. The autoimmune panel was negative apart from borderline high cardiolipin immunoglobulin G (IgG). Owing to recurrent bleeding, anticoagulation proved challenging, and she was ultimately managed with apixaban and clopidogrel. This case illustrates the synergistic impact of multiple mild thrombophilic factors and sickle cell trait, underscores the need to consider mesenteric venous thrombosis in abdominal emergencies, and highlights the clinical challenge of balancing thrombosis prevention with bleeding risk.

## Introduction

Venous thromboembolism (VTE) is uncommon in adolescents and, when present, often signals underlying thrombophilia or multisystem disease [1]. Cerebral venous sinus thrombosis (CVST) accounts for less than 1% of strokes but disproportionately affects young women and is frequently associated with prothrombotic states [2]. Mesenteric venous thrombosis is similarly rare yet carries significant morbidity due to the risk of intestinal ischemia and infarction [3].

Sickle cell trait (SCT), historically considered a benign condition, is increasingly recognized as a risk factor for thromboembolic events, particularly at unusual sites [4,5]. Proposed mechanisms include intermittent red cell sickling under hypoxic conditions, endothelial dysfunction, and a hypercoagulable state [6]. While SCT alone confers only modest risk, the presence of additional prothrombotic factors can amplify the likelihood of recurrent thrombosis [7].

Genetic polymorphisms such as Factor V R2 (H1299R), methylenetetrahydrofolate reductase (MTHFR C677T), and lymphotoxin-alpha (LTA C804A) are less well studied than classic variants such as Factor V Leiden or the prothrombin G20210A mutation, yet they may contribute to thrombotic risk, particularly when co-existing [8-10]. The interplay of multiple mild thrombophilic variants with SCT can produce a clinically significant prothrombotic phenotype, even when conventional thrombophilia panels appear negative [11].

Anticoagulation in such patients presents a therapeutic challenge. Warfarin is associated with substantial bleeding risk, particularly in those with underlying gastrointestinal pathology such as Helicobacter pylori gastritis [12]. Direct oral anticoagulants (DOACs) are increasingly employed but may not fully prevent recurrence [13]. In young women, menstrual bleeding adds another layer of complexity to long-term anticoagulation [14].

We present the case of an 18-year-old female with recurrent VTE at unusual sites, complicated by bleeding and multisystem involvement, in whom SCT and polygenic thrombophilia likely acted synergistically.

## Case presentation

**Figure 1:**
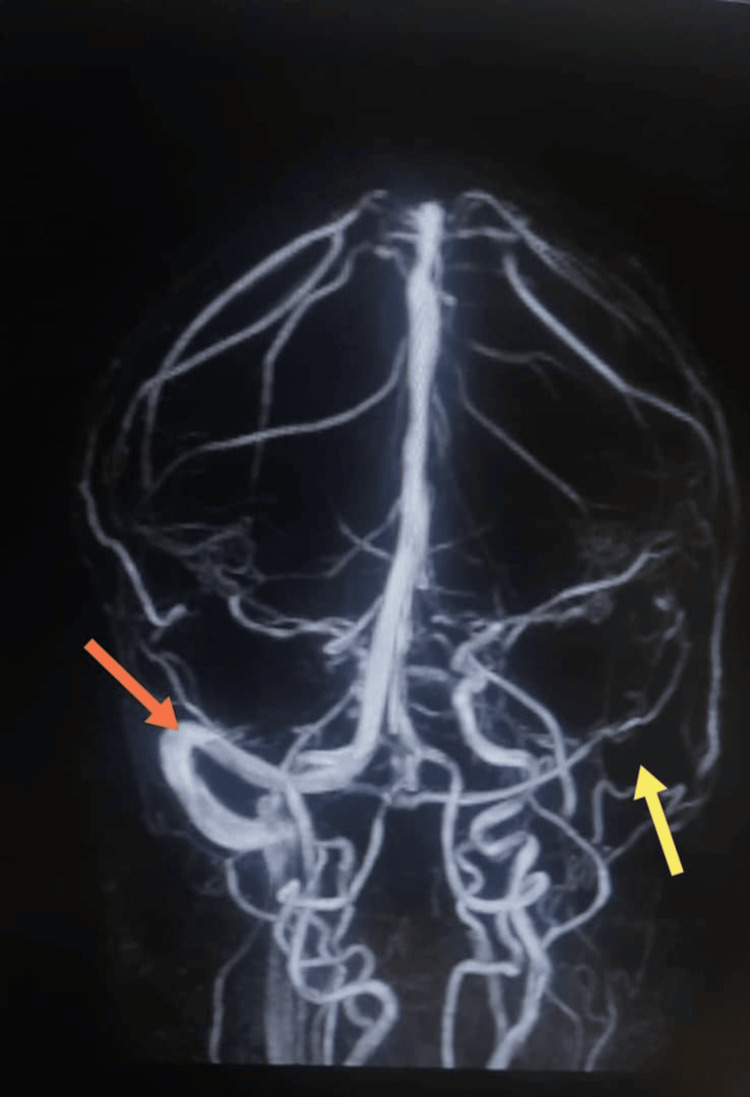
Brain MR venography (MRV) MRV demonstrating absence of flow in the left internal jugular vein, superior sagittal sinus, and left transverse sinus, consistent with cerebral venous sinus thrombosis (Yellow & Orange Arrows).

An 18-year-old female presented with severe headache. Magnetic resonance venography (MRV) of the brain demonstrated extensive CVST involving the sagittal and transverse sinuses (Figure 1). 

**Figure 2:**
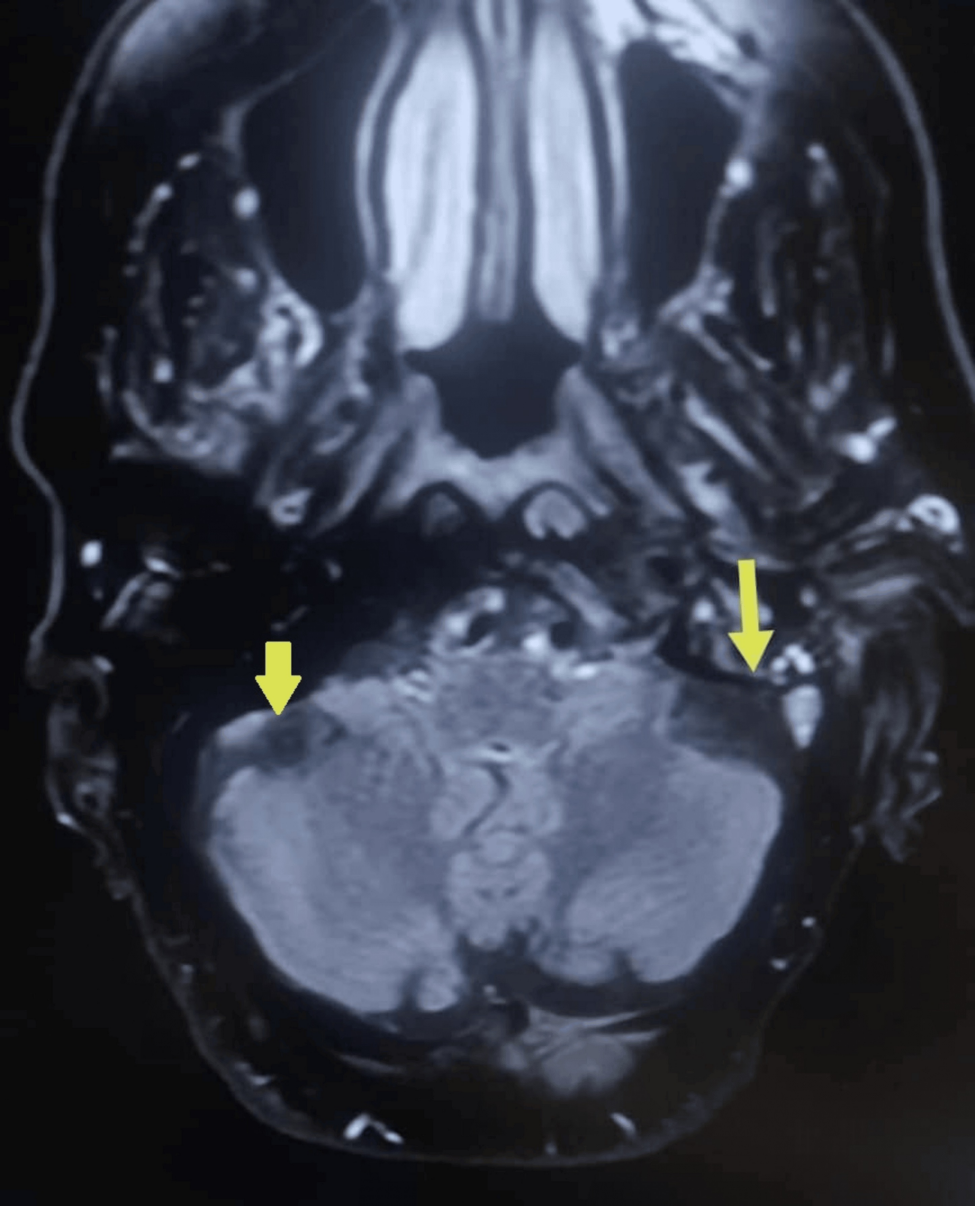
Magnetic resonance fluid-attenuated inversion recovery (FLAIR) sequence demonstrating increased signal intensity at the junction of the sigmoid sinus and internal jugular vein, suggestive of slow flow or thrombosis (Yellow Arrows).

Fluid-attenuated inversion recovery (FLAIR) MRI revealed increased signal intensity along the sinus-jugular confluence, consistent with slow flow or thrombus (Figure 2). 

Anticoagulation with warfarin was initiated. Within weeks, she developed massive upper gastrointestinal bleeding, with hemoglobin dropping to 5 g/dL, necessitating plasma transfusion and proton-pump inhibitor therapy. Following stabilization, anticoagulation was resumed, but she subsequently developed acute lower-limb deep vein thrombosis (DVT) despite therapeutic international normalised ratio (INR) levels.

**Figure 3:**
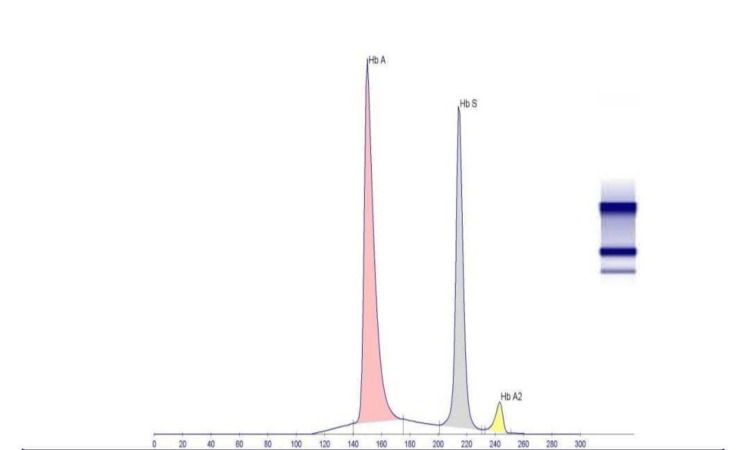
Graphical representation of hemoglobin (Hb) electrophoresis results illustrating decreased Hb A, elevated Hb A2, and the presence of Hb S.

Two thrombophilia and autoimmune panels were performed. Major markers, including anti-double-stranded DNA (dsDNA), antinuclear antibodies (ANA), and antineutrophil cytoplasmic antibodies (ANCA), were negative, with the exception of borderline cardiolipin IgG (15.6 units). Pelvic imaging was unremarkable; however, she was admitted twice for menorrhagia. Warfarin was switched to rivaroxaban, which stabilized her hemoglobin at 10 g/dL. Hemoglobin (Hb) electrophoresis revealed HbA 58.2%, HbS 38.3%, and HbA2 3.5%, consistent with sickle cell trait (Table 1, Figure 3). 

**Table 1:** Hemoglobin (Hb) electrophoresis results showing decreased Hb A, elevated Hb A2, and presence of Hb S.

Test name	Result	Normal rage	Unit	Last result
Hb A	58.2 L	96.7-98	%	57.8 L
Hb S	38.3			38.9
Hb A2	3.5 H	2-3.3	%	3.3

**Figure 4:**
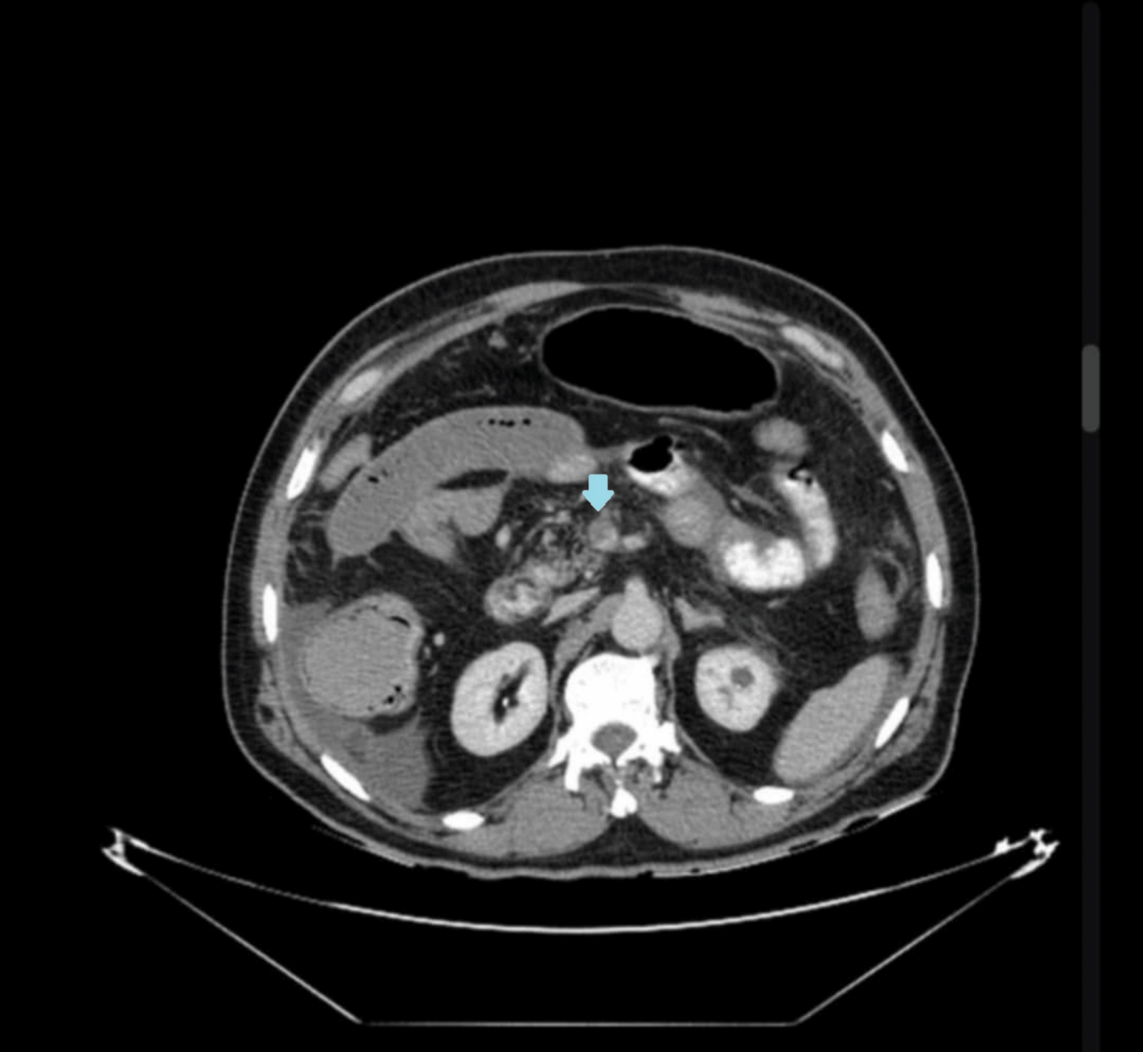
Contrast-enhanced abdominal CT (transverse view) Thrombosis of the superior mesenteric vein with adjacent bowel wall thickening; blue arrow indicates the thrombosed vein.

When anticoagulation was interrupted for two days, she developed another DVT, prompting initiation of enoxaparin. She later presented with severe epigastric pain radiating to the back and bilious vomiting. Contrast-enhanced CT of the abdomen demonstrated superior mesenteric vein thrombosis (MVT) with adjacent bowel wall thickening and hypoperfusion, consistent with venous ischemia (Figure 4). 

Diagnostic laparoscopy confirmed infarction of a jejunal loop with hemorrhagic peritoneal fluid, and resection was performed.

During this admission, upper gastrointestinal endoscopy revealed multiple ischemic-appearing gastric and lower esophageal ulcers with active bleeding. Gastric biopsy confirmed chronic active gastritis with Helicobacter pylori, for which eradication therapy was initiated. Laboratory evaluation showed microcytic anemia (Hb 10.6 g/dL, mean corpuscular volume (MCV) 68.3 fL, red cell distribution width (RDW) 18.9%), mild thrombocytopenia (141 × 10^9/L), elevated ESR (78-108 mm/hr), elevated CRP (45.3 mg/L), and hypertriglyceridemia (258 mg/dL) (Table 2). 

**Table 2:** Key laboratory values Selected laboratory results demonstrating microcytic anemia, mild thrombocytopenia, elevated ESR and CRP, and hypertriglyceridemia during the patient’s clinical course. ESR: Erythrocyte Sedimentation Rate, CRP: C-Reactive Protein

Parameter	Result	Reference Range / Note
Hemoglobin (Hb)	5 → 10.6 g/dL	12–16 g/dL (female)
Mean Corpuscular Volume (MCV)	68.3 fL	80–100 fL
Red Cell Distribution Width (RDW)	18.9%	11.5–14.5%
Platelet Count	141 × 10^9/L	150–450 × 10^9/L
ESR	78–108 mm/hr	0–20 mm/hr
CRP	45.3 mg/L	<10 mg/L
Triglycerides	258 mg/dL	<150 mg/dL
Hemoglobin Electrophoresis	HbS 35%	Normal: <5%
Homocysteine	5.6 μmol/L	5–15 μmol/L
Anticardiolipin IgG	15.6 units	<15 units (borderline)

A genetic thrombophilia panel identified heterozygous mutations in LTA (C804A), Factor V R2 (H1299R), and MTHFR (C677T), while Factor V Leiden (G1691A) was normal. Serum homocysteine was within the normal range at 5.6 μmol/L. These findings suggested a polygenic prothrombotic predisposition without hyperhomocysteinemia (Table 3). 

**Table 3:** Genetic thrombophilia panel Heterozygous mutations identified in LTA (C804A), Factor V R2 (H1299R), and MTHFR (C677T). Factor V Leiden (G1691A) was negative, and serum homocysteine levels were within the normal range. VTE: Venous thromboembolism

Gene / Variant	Zygosity	Clinical Significance / Notes
Factor V R2 (H1299R)	Heterozygous	Mild activated protein C resistance; modestly increased VTE risk
MTHFR C677T	Heterozygous	Usually minor effect if homocysteine normal; may contribute in polygenic context
LTA C804A	Heterozygous	Associated with pro-inflammatory and mild prothrombotic state
Factor V Leiden (G1691A)	Wild type / Normal	No increased risk from this allele

Her postoperative course was complicated by pancytopenia following hydroxyurea therapy for cytoreduction, which was subsequently discontinued. Chest CT revealed patchy subpleural reticulations and two small mediastinal/para-aortic lymph nodes (1 × 4 cm), interpreted as reactive. Abdominal ultrasound demonstrated medullary sponge kidney with nephrocalcinosis.

Later, she developed upper-limb swelling, with ultrasound showing soft tissue edema, suspicious for early cellulitis or osteomyelitis. She improved with cefixime and conservative therapy. Given recurrent thrombosis during interruptions of anticoagulation and prior life-threatening bleeding, a tailored regimen of apixaban plus clopidogrel was initiated.

To provide a clear overview of the sequence of clinical events, investigations, and management, a structured timeline summarizing key milestones is presented in Table 4.

**Table 4:** Clinical timeline of key events and management DVT: deep vein thrombosis, LMWH: low-molecular-weight heparin

Timepoint	Clinical Event	Investigations	Management
Presentation (Day 0)	Headache, seizures → diagnosed with cerebral venous sinus thrombosis (CVST)	MR venography showing sagittal sinus and internal jugular vein thrombosis	Warfarin initiated
Day 15	Massive upper GI bleeding	Endoscopy: ischemic gastric & esophageal ulcers, *H. pylori* gastritis	Warfarin discontinued, supportive therapy
Week 4	New left leg swelling	Ultrasound: femoral DVT	Temporary anticoagulation with LMWH
Month 2	Abdominal pain	CT: superior mesenteric vein thrombosis with bowel infarction	Surgical resection + anticoagulation
Month 3	Recurrent bleeding on warfarin	—	Transitioned to apixaban + clopidogrel
Ongoing follow-up	No further major bleeding, stable on therapy	—	Indefinite anticoagulation planned

## Discussion

This patient demonstrates a striking clustering of venous thromboembolic events - CVST, recurrent limb DVT, and MVT - at an unusually young age, in the absence of malignancy or sustained provoking factors. CVST is rare but classically associated with prothrombotic states; early anticoagulation is standard even in the presence of intracranial hemorrhage, although subsequent management must be individualized when bleeding risk is high [1].

Sickle cell trait (SCT) approximately doubles the risk of VTE, particularly pulmonary embolism, compared with noncarriers. While the absolute risk remains modest, SCT can synergize with other inherited and acquired factors to produce clinically significant thrombophilia [2,3].

Inherited thrombophilias identified in this patient - Factor V R2 (H1299R), MTHFR C677T, and LTA (C804A) - each individually confer only a small increase in thrombotic risk, but combined variants may have additive effects, particularly in the presence of inflammatory triggers (e.g., H. pylori gastritis) and perioperative stress [4-6]. Factor V R2 is associated with mild activated protein C resistance and decreased factor V levels, conferring approximately a two-fold risk of VTE; the absence of Factor V Leiden does not negate this effect [5]. The MTHFR C677T variant is of uncertain significance when homocysteine is normal, as in this patient, but may contribute marginally in polygenic contexts [6]. The LTA C804A polymorphism has been linked to enhanced vascular inflammatory signaling, plausibly acting as a weak prothrombotic cofactor [10].

Mesenteric venous thrombosis can lead to venous outflow obstruction, bowel wall edema, hemorrhage, and infarction; timely recognition is essential to prevent transmural necrosis and peritonitis [7,8]. The ischemic gastric and distal esophageal ulcers observed endoscopically likely reflect venous congestion rather than classic acid-peptic disease, a rare but recognized manifestation in extensive splanchnic venous thrombosis [7,8]. Concomitant H. pylori infection likely exacerbated mucosal injury and bleeding risk, warranting eradication therapy to reduce rebleeding and anemia [9]. Notably, Helicobacter pylori infection has also been associated with prothrombotic states and thrombocytopenia, possibly through chronic inflammation and platelet activation. In this case, the delayed eradication may have contributed to the persistence of a hypercoagulable milieu, adding further complexity to the patient’s thrombotic presentation [15].

Therapeutically, this case underscores the anticoagulation dilemma: recurrent thrombosis occurred even during brief interruptions, yet warfarin precipitated life-threatening gastrointestinal hemorrhage. Population data suggest differential gastrointestinal bleeding profiles among anticoagulants, with rivaroxaban and high-intensity warfarin generally carrying higher risk than apixaban; individual risk assessment and shared decision-making remain paramount [11]. Heavy menstrual bleeding is a common complication in young women on anticoagulation and requires proactive management, including hormonal therapy or levonorgestrel intrauterine device placement in collaboration with hematology and gynecology [12]. Ultimately, an apixaban-based regimen with adjunct antiplatelet therapy was selected to balance thrombotic risk against bleeding, alongside meticulous gastrointestinal protection and H. pylori eradication. Indefinite anticoagulation is often appropriate after unprovoked or recurrent VTE when bleeding risk is controlled [4].

Other findings - including hydroxyurea-induced pancytopenia, soft tissue infection, medullary sponge kidney, and reactive lymphadenopathy - were managed according to standard principles. Hydroxyurea-related myelosuppression mandates dose interruption or discontinuation with monitoring [13]. Medullary sponge kidney is typically incidental, associated with nephrocalcinosis and stone risk rather than thrombosis, and managed conservatively [14]. The transient limb infection resolved with oral antibiotics and supportive care.

Compared with previously reported cases of venous thromboembolism in adolescents, this case is unique due to the combination of multiple mild prothrombotic genetic variants, sickle cell trait, and an inflammatory trigger in the form of H. pylori infection, leading to recurrent and multisystem thrombotic events including CVST, DVT, and mesenteric venous thrombosis. While SCT alone has been associated with modestly increased VTE risk, the synergistic effect of polygenic thrombophilia and delayed eradication of H. pylori in this patient underscores a rare and clinically challenging phenotype. This highlights the importance of comprehensive evaluation and individualized management in similar complex presentations.

In summary, this case illustrates how multiple “lesser-known” prothrombotic factors - SCT combined with polygenic thrombophilia - can collectively produce a high-risk thrombotic phenotype, particularly in the presence of inflammatory triggers. Clinicians should anticipate rapid recurrence during anticoagulation interruption, treat modifiable contributors aggressively, and tailor antithrombotic therapy with careful attention to bleeding-prone sites [2,4,7-9,11].

## Conclusions

Sickle cell trait, polygenic thrombophilia, and inflammatory triggers can synergistically produce recurrent and severe venous thromboembolism in young patients. The challenge of balancing anticoagulation with life-threatening bleeding underscores the importance of individualized therapy. In this case, recurrence was ultimately controlled with apixaban-based anticoagulation combined with gastrointestinal protection and H. pylori eradication, highlighting the need for multidisciplinary management. Clinicians should maintain a high index of suspicion for mesenteric venous thrombosis in patients presenting with abdominal pain and known thrombophilia, manage inflammatory contributors such as H. pylori aggressively, and anticipate rapid recurrence during interruptions of anticoagulation. Long-term follow-up is essential, as indefinite anticoagulation is often required to prevent further thrombotic events in high-risk individuals.
